# Traditional cooking practices and preferences for stove features among women in rural Senegal: Informing improved cookstove design and interventions

**DOI:** 10.1371/journal.pone.0206822

**Published:** 2018-11-20

**Authors:** Laura G. Hooper, Yakou Dieye, Assane Ndiaye, Aldiouma Diallo, Coralynn S. Sack, Vincent S. Fan, Kathleen M. Neuzil, Justin R. Ortiz

**Affiliations:** 1 Department of Medicine, University of Washington, Seattle, Washington, United States of America; 2 PATH, Dakar, Senegal; 3 Vitrome Institute de Recherche pour le Developpement, Dakar, Senegal; 4 Center for Vaccine Development and Global Health, University of Maryland School of Medicine, Baltimore, Maryland, United States of America; Tulane University School of Public Health and Tropical Medicine, UNITED STATES

## Abstract

Nearly half the world’s population burns solid fuel for cooking, heating, and lighting. The incomplete combustion of these fuels is associated with detrimental health and environmental effects. The design and distribution of improved cookstoves that increase combustion efficiency and reduce indoor air pollution are a global priority. However, promoting exclusive and sustainable use of the improved stoves has proved challenging. In 2012, we conducted a survey in a community in rural Senegal to describe stove ownership and preferences for different stove technologies. This report aims to describe local stove and fuel use, to identify household preferences related to stove features and function, and to elicit the community perceptions of cleaner-burning stove alternatives with a focus on liquid propane gas. Similar to many resource-limited settings, biomass fuel use was ubiquitous and multiple stoves were used, even when cleaner burning alternatives were available; less than 1% of households that owned a liquid propane stove used it as the primary cooking device. Despite nearly universal use of the traditional open fire (92% of households), women did not prefer this stove when presented with other options. Propane gas, solar, and improved cookstoves were all viewed as more desirable when compared to the traditional open fire, however first-hand experience and knowledge of these stoves was limited. The stove features of greatest value were, in order: large cooking capacity, minimal smoke production, and rapid heating. Despite the low desirability and smoke emisions from the traditional open fire, its pervasive use, even in the presence of alternative stove options, may be related to its ability to satisfy the practical needs of the surveyed cooks, namely large cooking capacity and rapid, intense heat generation. Our data suggest women in this community want alternative stove options that reduce smoke exposure, however currently available stoves, including liquid propane gas, do not address all of the cooks’ preferences.

## Introduction

Household air pollution is the single most important environmental risk factor for disease worldwide [1]. An estimated 3 billion people, nearly 50% of the global population, uses solid fuels for cooking, heating and, and the vast majority live in low- and middle-income countries (LMICs) [2]. The incomplete combustion of solid and biomass fuel generates volatile gases and respirable particulate matter that are associated with both acute and chronic disease and increased risk of death [3–5]. The World Health Organization (WHO) estimates that 4.3 million premature deaths were due to exposure to household air pollution in 2012 alone [6]. Women and children disproportionately experience higher household air pollution exposure due to women’s often traditional roles doing indoor work, cooking, and childcare [1,7].

International efforts to reduce indoor air pollution from cookstoves and associated health effects in LMICs have been championed by the WHO, United Nations and the World Bank and directed by multinational partnerships such as The Global Alliance for Clean Cookstoves (GACC) [8–10]. Until recently these efforts have focused on the development and distribution of improved cookstoves (ICS) in LMICs [8,9]. ICS technology is characterized by improving fuel combustion efficiency and reducing household air pollution through redesign of the simple biomass fuel-burning cookstoves. However, these efforts have disappointed with poor uptake and failure to demonstrate reduction in household air pollution levels or health effects [11,12]. As a result, the field has begun to shift focus to cleaner fuel alternatives with a particular emphasis on the promotion of liquid propane gas (LPG), biogas and electricity [13,14]. Cost and lagging infrastructure are broadly recognized barriers, but knowledge gaps and perceptions of these technologies at the community level must also be addressed to optimize adoption and use. There is no universal strategy for a successful clean stove campaign; decisions regarding stove adoption seem variably influenced by a complex interplay of factors including cultural appropriateness, household preferences, incentives, and socioeconomics [15,16]. Understanding community-level preferences and perceptions regarding improved stoves is one component to solving the puzzle of successful clean stove adoption.

The aim of this research study is to describe traditional cooking practices and fuel use, to identify perceived preferences and disadvantages of traditional stoves, and to describe community opinions of LPG and ICS for a rural community in Senegal. This study considers stove features and functionality that are of greatest value to rural Senegalese cooks. Knowledge of these specific preferences and beliefs is important for informing future stove intervention trials.

## Materials and methods

### Study setting

The study was performed in the Niakhar area in the Fatick Health District of Western Senegal, located approximately 135km east of Dakar. The population is predominantly ethnic Sereer (97%) and Muslim (77%) [17]. Sereer is the primary language for the region while French is the national language of Senegal. Niakhar is an arid region that borders the Sahel. Agriculture is the foundation for the economy, with most households growing millet and groundnuts. Formal education levels are low among residents with 75% of women between ages 15–24 never attending primary school [17]. Nuclear families live within households that cluster together with the extended patrilineal family to form a larger compound. Women within this extended family structure frequently share the food preparation duties by cooking for more than one household within their compound.

Thirty villages in the Niakhar region participate in demographic surveillance that has been ongoing since 1983 [17]. The demographic surveillance system (DSS) is organized by the Institut de Recherche pour le Developpement (IRD) and is a member of the INDEPTH Network that supports health and population research in Africa and Asia.

### Study design

**Fig 1:**
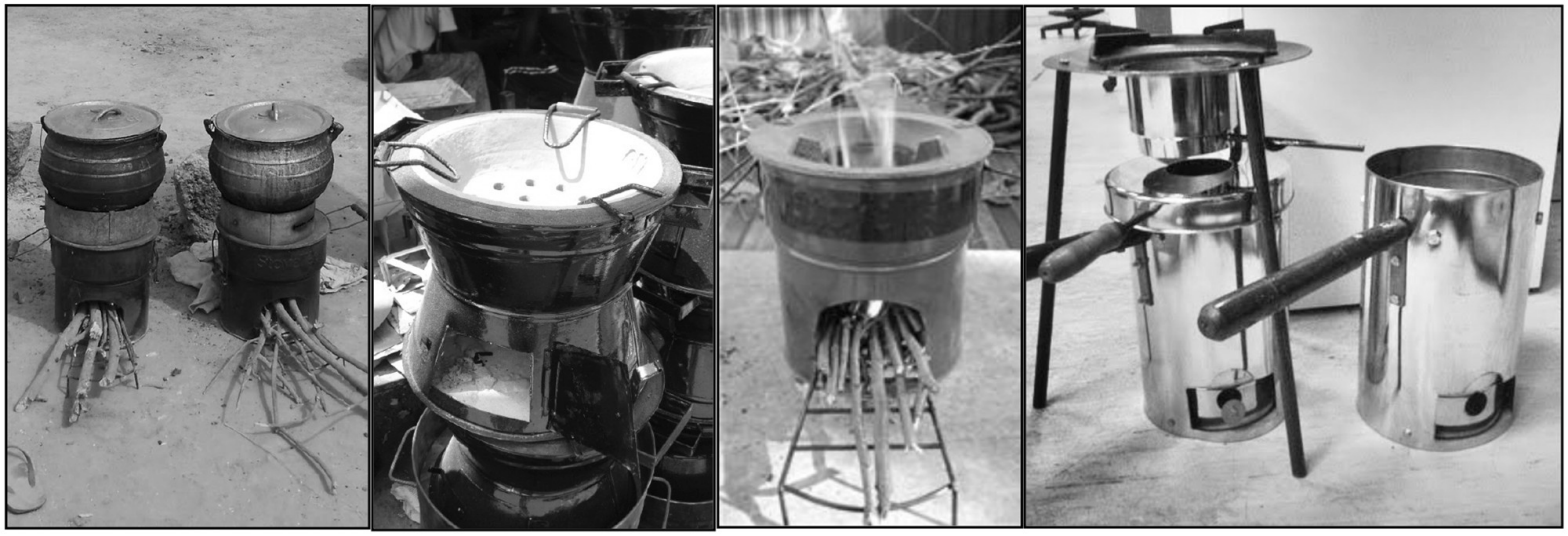
Composite image of various improved cookstoves shown to respondents when asked for their opinions about improved cookstoves.

The study was a cross-sectional survey of rural Senegalese women regarding their household cooking habits and the perceived benefits and shortcomings of traditional and improved cookstoves. The survey was adapted from previously published surveys related to respiratory health, indoor smoke exposure, and fuel use [18,19]. In 2009, we visited 15 households in the study area and piloted the survey [20]. During our pilot study, high levels of cooking smoke were primary complaints about current cookstoves during home visits and in the pilot study survey which asked about respiratory symptoms after asking stove and fuel-related questions. The study team included researchers from the University of Washington, PATH, and local investigators with over 30 years of experience living and working in the Niakhar area. Subsequent revisions to the instrument were made by the study team based on lessons learned from the pilot study and feedback from participants. The revised survey was administered in 2012 and included questions about current household stove and fuel use patterns, beliefs about the health effects of biomass smoke exposure, and preferences regarding stove features and functionality. The interviewers were trained field workers who had no affiliation with local health institutions. The survey was conducted in a neutral manner and no health objective was specified, however the questions on childhood respiratory symptoms preceded the survey about cookstoves. When asked about stove features, participants were presented with a pre-coded list from which to select their preferences. Survey questions referenced a broad array of stove types, including the traditional three-stone open fires, LPG stoves, solar cooking technology, enclosed stoves with outdoor venting chimneys, charcoal pots, and ICS. Survey questions about ICS specifically referenced the rocket stove, side-feed chamber style and the gasifier (or top lit updraft) style; participants were shown a composite set of images of these ICS designs when asked about ICS as a general stove category (Fig 1).

Households within the four most populous DSS villages were surveyed; Diohine, Ngayokhem, Ngangarlame, and Toucar. These villages were selected to maximize the number of study subjects by allowing field workers to contact the greatest number of households within a geographic area given available resources. The largest villages were more consistently accessible during the rainy seasons and including them allowed our field workers to contact the greatest number of households within a defined geographic area. This survey was co-administered with a survey on asthma symptoms in children [21]. All households within each village with at least one child aged five through eight years old (the inclusion criteria for the asthma portion) were included. As all eligible households in the four study villages were included in the survey, we did not need to perform statistical sampling to make inference about a larger group. Households with age-qualifying children were identified using the DSS database and approached for participation by trained field workers. The woman who identified as the primary caregiver for the children was identified and asked to participate. It was confirmed that this woman also shared in cooking responsibilities for the household, consistent with cultural norms for this community. Participation was limited to women, given that women are the primary cooks in Senegal. Public health experts from the Niakhar area participated in survey development to ensure questions were culturally appropriate and relevant to respondents within the local environment. Ongoing demographic surveillance has indicated that persons in the largest villages are very similar with regards to socioeconomic status and other demographic characteristics to persons living elsewhere in the DSS, so we determined that the decision made for feasibility did not sacrifice generalizability to other parts of the area. The survey was translated from English to French by a bilingual collaborator then back-translated to English to confirm content conservation. Given the low literacy rates in Niakhar, trained field workers, fluent in both French and Sereer, verbally administered the survey to respondents.

### Ethical approval

This study received ethical approval by the University of Washington Institutional Review Board and the Senegal national ethics committee, Comité National des Etudes et Recherches en Santé (CNERS). Acceptance for the study was obtained from community leaders, local religious leaders, and the administrators of health facilities in Niakhar. In accordance with cultural norms, field workers received verbal permission from the heads of each household prior to obtaining written informed consent from survey respondents.

### Data analysis

Survey data were entered into REDCap (Research Electronic Data Capture), a secure, web-based, data capture application hosted at the University of Washington [22]. Descriptive analyses are reported for participant responses. Multivariable logistic regression was used to model associations between participant characteristics and first-choice stove preference. Only the top three stove choices were modeled to minimize multiple comparisons and because they were available in the study area enabling familiarity by respondents and reliability of results. Covariates included in the model, selected *a priori*, based on investigators’ review of the relevant literature. These were age, socioeconomic status, family size for meal preparation, stove location, and respiratory symptoms reported by cook and children. Measures of association are reported as odds ratios (OR). In the logistic regression model of general stove characteristics predicting LPG preference, the issue of multiple predictors was addressed using backward stepwise regression with variables eliminated using a significance threshold of 0.2. LPG stove characteristics (as compared to primary stove) included in the initial model were: reduced time collecting fuel, reduced smoke production, increased cooking speed, ability to slow cook (“simmer”), health benefits, ease of adjusting heat level, improved taste of food, and reduced risk of burns to children. Health benefits and improved taste of food were excluded from the final model via the backward stepwise procedure. For analyses of predictors of stove preference, we focused on characteristics that were associated with preferring LPG technology, given the international interest in promoting LPG use. Additionally, Niakhar has access to LPG stoves and experience with their use leading to more informed opinions about preference for this stove. Too few respondents selected ICS as a preferred stove type to allow for similar analyses. As no solar stoves are in use in this community we did not analyze this preference given a concern that opinions about valuable features would have limited foundation in practical knowledge of this technology. In all analyses, p-values < 0.05 were considered statistically significant. All statistical analyses were performed using Stata Statistics Software version 14 (StataCorp LP, College Station, TX).

## Results

### Demographic information

A total of 1103 women from separate households completed the survey. The mean age of respondents was 36 years (standard deviation, SD = 10) (Table 1). There was an average of two households in each compound, however, 29% of compounds were comprised of 3 or more households. On average, there were 7.5 children (SD = 3.6) per household. One third of respondents cooked for 7–10 people daily, while 61% reported cooking for 11 or more people per day.

**Table 1:** Characteristics of study participants and households in Niakhar region of Senegal.

	Total (N = 1103)	
	Count	(%)
Participant Characteristics		
Age, mean [SD]	35.7	[10]
Female	1,103	(100)
Smokes tobacco	4	(<1)
Relationship to children in household		
Mother	890	(81)
Grandmother	105	(10)
Sister	10	(1)
Aunt	47	(4)
No response given	50	(5)
Household (HH) Characteristics		
No. of HH in compound, mean [min, max]	2.3	[1,11]
No. of children per HH, mean [SD]	7.5	[3.6]
No. of people respondent cooks for per day		
4–6	68	(6)
7–10	362	(33)
11 or more	671	(61)
Assets		
TV	136	(12)
Electricity	137	(12)
Access to running water or bore hole	301	(27)
Mobile phone	1,049	(95)
No response given	27	(2)
Building material		
Mud bricks or mud thatch	973	(89)
Cement bricks	105	(10)
Zinc/Corrugated metal	10	(1)
Compound with resident smoker	654	(59)
Types of stoves owned by the household		
Traditional	1014	(92)
Coal Pot	63	(6)
Liquid propane gas	287	(26)
Improved cook stove (ceramic insulated, side-feed design)	238	(22)
Solar stove	0	(0)
No response given	29	(3)
Households that own 2 or more stoves	452	(41)
Primary Stove Used in Household in dry season^a^		
Traditional (3-stone or metal tripod)	753	(68)
Coal Pot	143	(13)
Improved Cook Stove (ceramic insulated, side-feed design)	20	(2)
Liquid propane gas	4	(<1)
No response given	183	(17)
Household Health Measures		
Child in HH with wheeze	123	(12)
Child in HH with nocturnal cough	262	(24)
Child in HH with severe asthma symptoms	78	(7)
Cook has difficulty breathing after cooking	163	(15)
Cook has wheezing after cooking	110	(10)
Cook has cough with phlegm	167	(15)

Due to incomplete responses to some questions, not all percentages add to 100.

^a^ Primary stove use reported for the dry season; rainy season figures are comparable (Traditional: 69%, Coal Pot: 13%, ICS: 2%, LPG: <1%, missing 17%). Item or unit non-response is reported when non-response exceeds 2%.

Households were generally of a traditional hut design with 973 (90%) constructed of mud bricks or a composite of mud and sticks/straw. Access to a community water tap was reported for 301 (27%) households. Electricity was reported in 137 (12%) households. While 12% of households reported having a television, 1,049 (95%) owned a mobile phone.

### Current stove and fuel use patterns

**Fig 2:**
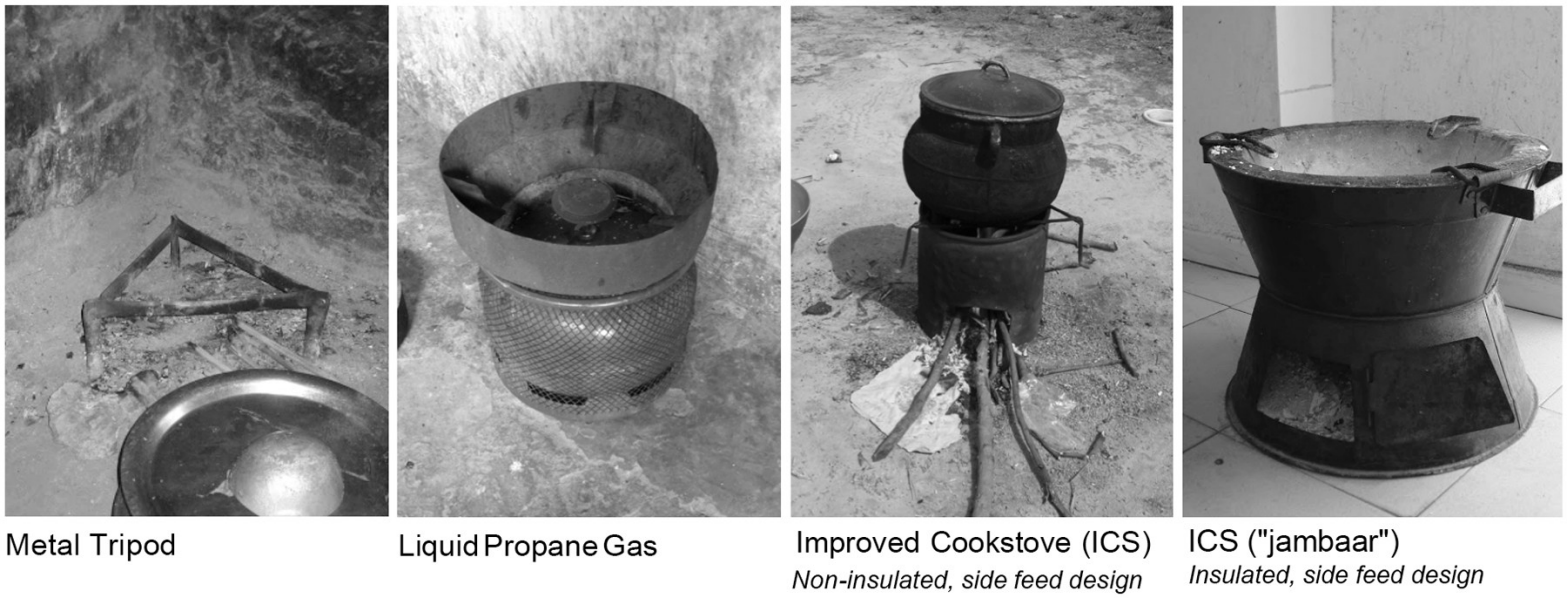
Images of cookstoves used in survey area.

The traditional cooking method is an open-fire stove created by arranging three stones or a metal tripod on the floor of the hut (Fig 2). The majority of households, 1,014 (92%), use this type of traditional tripod stove arrangement, although many households (41%) own 2 or more types of stoves (Table 1). Over two-thirds of households, 753 (68%) in the dry season and 756 (69%) in the rainy season, relied on a traditional tripod stove as their primary cooking method. Liquid propane gas (LPG) stoves were used in 287 (26%) households, however <1% reported using LPG primarily. Almost all households used a combination of biomass fuel in the form of wood (n = 1,100; 99%), crop residue (n = 1,079; 98%) or dung cakes (n = 992; 90%) for at least some of their cooking.

The transition from rainy to dry season did not alter cooking habits; the majority of households continued to use traditional tripod stoves and biomass fuel in both seasons. Seasonal variation in the type of biomass fuel was observed; during the rainy season 698 households exclusively burned wood and only one household burned dried dung cakes, while during the dry season, 353 households burned wood and 300 reported burning dung. During the rainy season, 837 (76%) women cooked indoors. During the dry season, 480 (44%) continued to cook in enclosed or semi-enclosed spaces while 452 (41%) reported cooking outdoors or in open kitchen structures with no walls.

### Ideal stove type and predictors of stove preference

When asked what type of stove they most desired (LPG, ICS, wood stove with chimney, electric, solar, or traditional tripod stove), irrespective of cost for either the stove or fuel, 380 (35%) participants selected LPG stoves and 313 (28%) chose solar-powered stoves. Wood-stoves with a venting chimney were the preferred stove type for 274 (25%) of participants. An ICS was preferred by only 32 (3%) respondents. Only 14 (1%) participants identified the traditional tripod stove as the most preferred design. Among the 287 participants who already owned an LPG stove, 43% identified a solar stove as their ideal stove choice. Notably, none of the 1,103 surveyed households owned a solar-powered stove; they are not known by our study team to be used in this community.

Associations between respondent characteristics and top stove preference are presented in Table 2. Increasing age was associated with decreased preference for LPG and increased preference for a stove with an outdoor venting chimney when compared to all other stove choices (OR for LPG = 0.98, 95% CI = 0.97–0.99, *p* = 0.004, OR for chimney stove = 1.02, 95% CI = 1.01–1.04, *p*<0.001). Cooking indoors was associated with preference for a chimney-vented stove (OR = 1.57, 95% CI = 1.00–2.45, p = 0.05). Respondents with respiratory symptoms (cough, phlegm, or wheeze) were more likely to prefer a chimney-vented stove (OR = 1.40, 95% CI = 1.00–1.94, *p* = 0.049), but less likely to prefer solar (OR = 0.62, 95% CI = 0.43–0.89, *p* = 0.01). Wheezing among children in the household was not associated with stove preference. The number of persons to cook for was not predictive of preferred stove type.

**Table 2:** Associations (odds ratios) between respondent characteristics and first choice of most desired stove type.

	Top Stove Choice^a^ n = 990								
	Liquid Propane Gas			Stove w/ Chimney			Solar		
Characteristic	OR	(95% CI)	p-value	OR	95% CI	p-value	OR	95% CI	p-value
Age, years	0.98	0.97–0.99	0.004	1.03	1.01–1.04	<0.001	0.99	0.97–1.00	0.059
Socioeconomic status^b^									
Low	Referent			Referent			Referent		
High	1.08	0.83–1.42	0.562	0.48	0.35–0.67	<0.001	1.03	0.76–1.39	0.863
No. people to cook for/day									
4–6	Referent			Referent			Referent		
7–10	0.87	0.49–1.53	0.626	0.89	0.50–1.70	0.715	1.08	0.59–1.97	0.807
11 or more	1.00	0.58–1.72	0.991	1.33	0.71–2.49	0.365	0.67	0.37–1.20	0.176
Cooking location, rainy season									
Outside	Referent			Referent			Referent		
Enclosed kitchen	0.76	0.53–1.08	0.133	1.57	1.00–2.45	0.050	0.75	0.51–1.10	0.145
Respiratory symptoms of cook	1.08	0.80–1.46	0.610	1.40	1.00–1.94	0.049	0.62	0.43–0.89	0.010
Child with wheeze in household	0.93	0.66–1.31	0.676	1.11	0.76–1.62	0.594	0.87	0.59–1.29	0.488

Multivariable logistic regression model with each outcome (top stove choice) compared to all participants without that outcome. The total number of participants with non-missing data on all covariates was 990 for each outcome analysis. Model adjusted for all characteristics as shown in table. Inclusion of village as a variable did not alter the direction or significance of the outcomes.

^a^ Each outcome is mutually exclusive; each participant was only able to select one stove as their first-choice selection. Each stove preference listed is compared to all other stove options (liquid propane gas, improved cookstove, wood stove with chimney, electric, solar, or traditional tripod stove)

^b^ Socioeconomic status defined by household building materials and household assets. Higher SES households used cement blocks and corrugated zinc as building materials and possessed a television and/or had access to electricity. Lower SES households used mud bricks, mud and sticks, and straw as building materials and lacked electricity and television

### Desirable stove features

**Fig 3:**
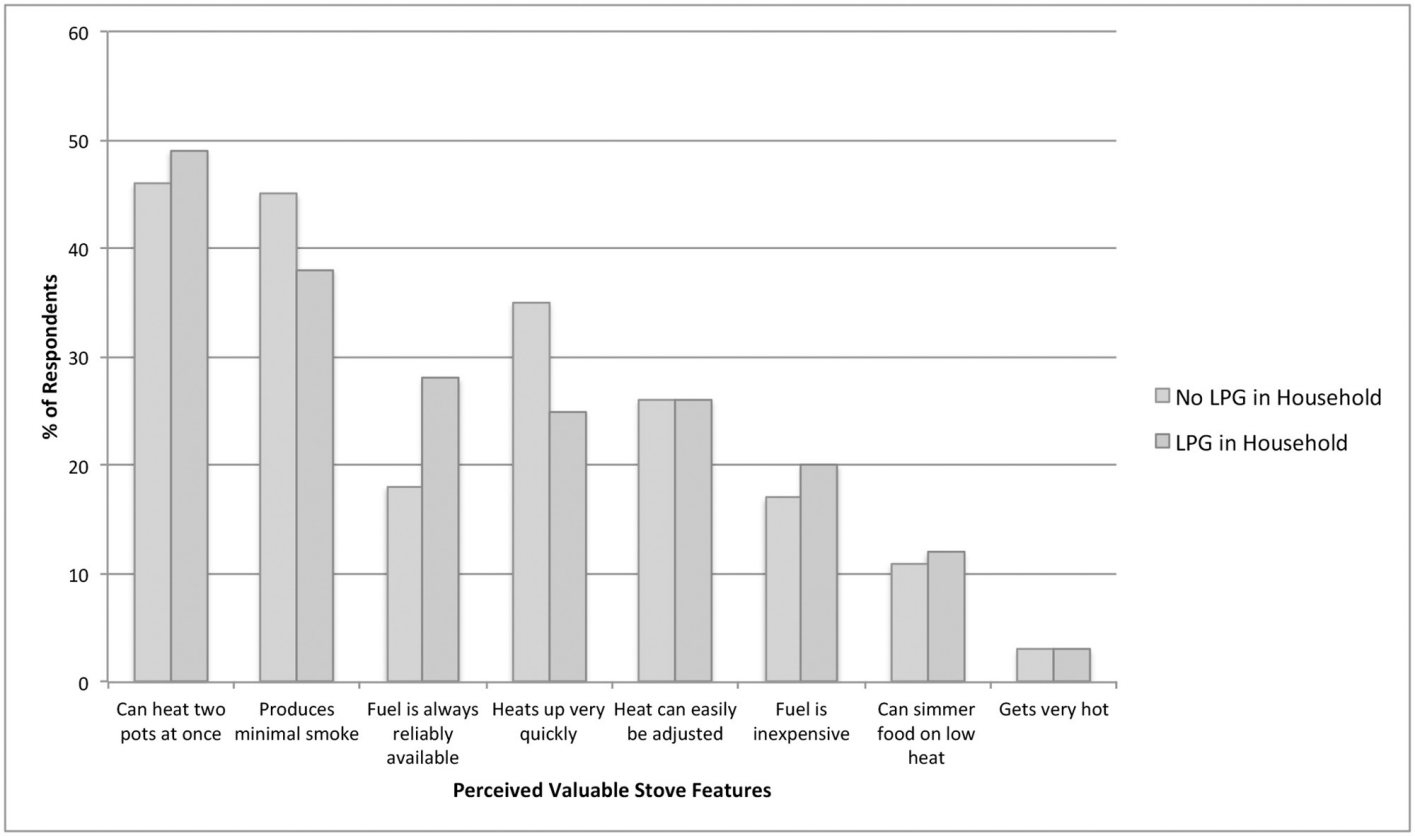
General stove features considered most valuable, stratified by households with and without an LPG stove.

When asked about stoves in general, the stove features participants considered most important were cooking capacity (n = 514; 47%), minimal smoke production (n = 475; 43%), and heating efficiency (n = 355; 32%) (Fig 3). Heating efficiency was more important to respondents from households that did not own LPG than those that did. In comparison, households that owned LPG were more likely to value fuel availability than non-LPG households. Features such as precise temperature control (n = 119; 11%), and fuel availability (n = 227; 21%) and expense (n = 198; 18%), were less important to respondents than minimizing smoke production.

Among the 380 respondents who identified LPG as their most desired stove, reduced smoke emission was the most frequently cited reason for their choice (n = 189, 50%). Among women who preferred a solar powered stove, time saved in fuel collection was the most frequently identified feature that influenced their decision (n = 207, 66%). For participants who wanted a wood stove with an outdoor venting chimney, the belief that it cooked food faster than their current stove was the most often cited reason (n = 154, 56%).

In the multivariable backward stepwise logistic regression model of stove features associated with LPG preference, participants who identified reduced smoke emission (OR = 1.9, 95% CI = 1.3–2.7, p<0.01), precise temperature control (OR = 2.2, 95% CI = 1.5–3.3, p<0.001), or increased cooking speed (OR = 1.8, 95% CI = 1.3–2.4, p<0.01) as important stove features, were more likely to prefer LPG to other stoves. LPG was less likely to be the preferred stove type among participants who placed the highest value on minimizing time collecting fuel (OR = 0.5, 95% CI = 0.4–0.8, p<0.001) and safety from burns for young children (OR = 0.4, 95% CI = 0.3–0.7, p<0.001).

### Beliefs about ICS and LPG

**Fig 4:**
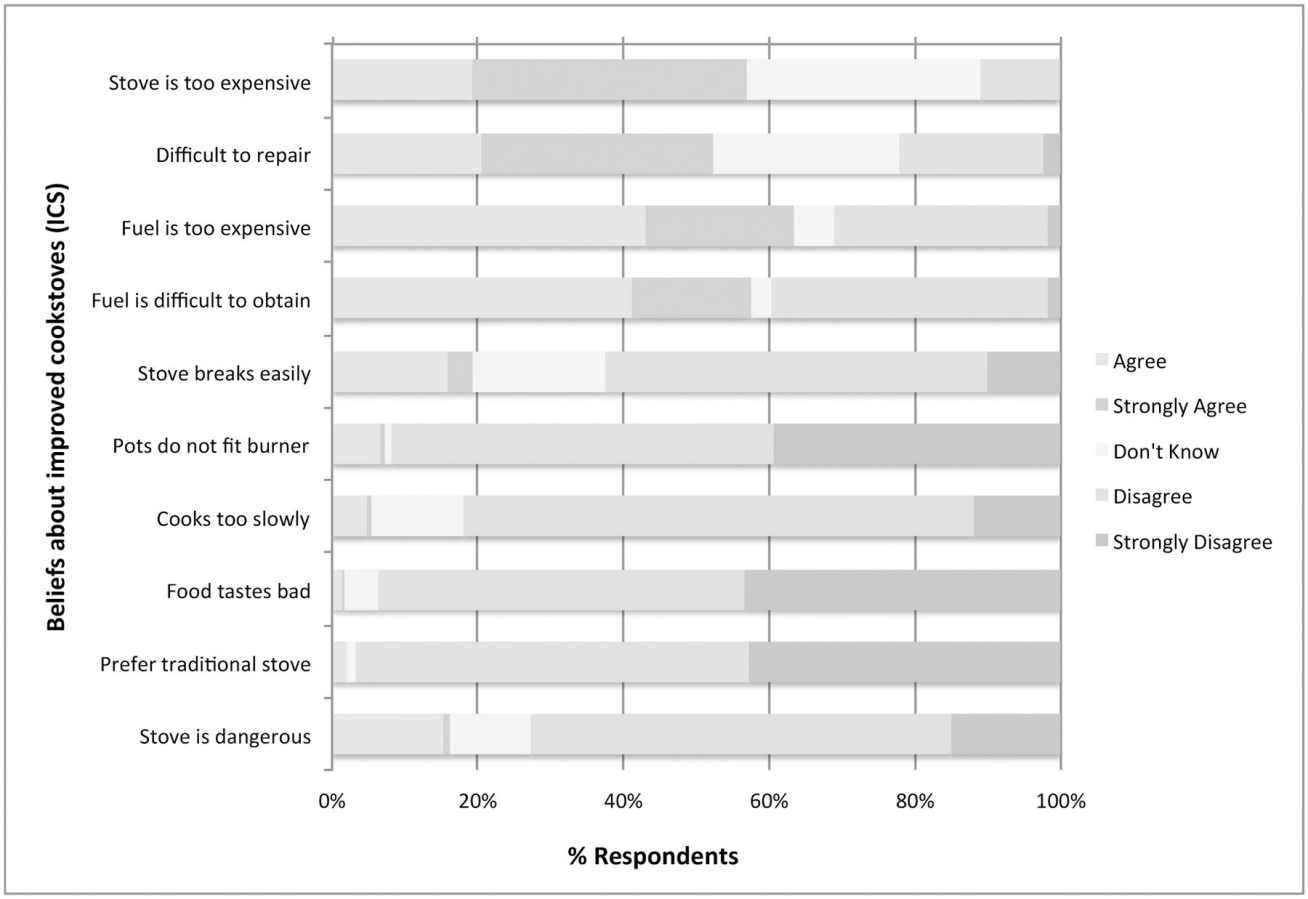
Likert scale presentation of perceptions of improved cookstoves (ICS).

Although less than 1% of households were reported to have an ICS, almost all respondents (n = 1093, 99%) believed ICS were superior to traditional stoves. No one stated they would prefer cooking on a traditional stove to an ICS. When asked specifically about the perceived merits and drawbacks of ICS, they were believed to have good cooking efficiency (82% disagreed with statement “these stoves take too long to cook food,” n = 905) and generally seen as durable (63% disagreed with statement “stoves break too easily,” n = 690) (Fig 4). The majority (92%) of respondents believed the available ICS were well designed to accommodate the pot sizes typical of rural Senegalese households. Over three-quarters of respondents (n = 848, 77%) felt food cooked with ICS tasted the same or better than food cooked with a traditional stove. Cost was a perceived weakness for ICS, with 57% (n = 629) reporting the ICS were too expensive to purchase. While ICS stoves were viewed as durable, 52% (n = 576) felt they were difficult or costly to repair if they did break.

**Fig 5:**
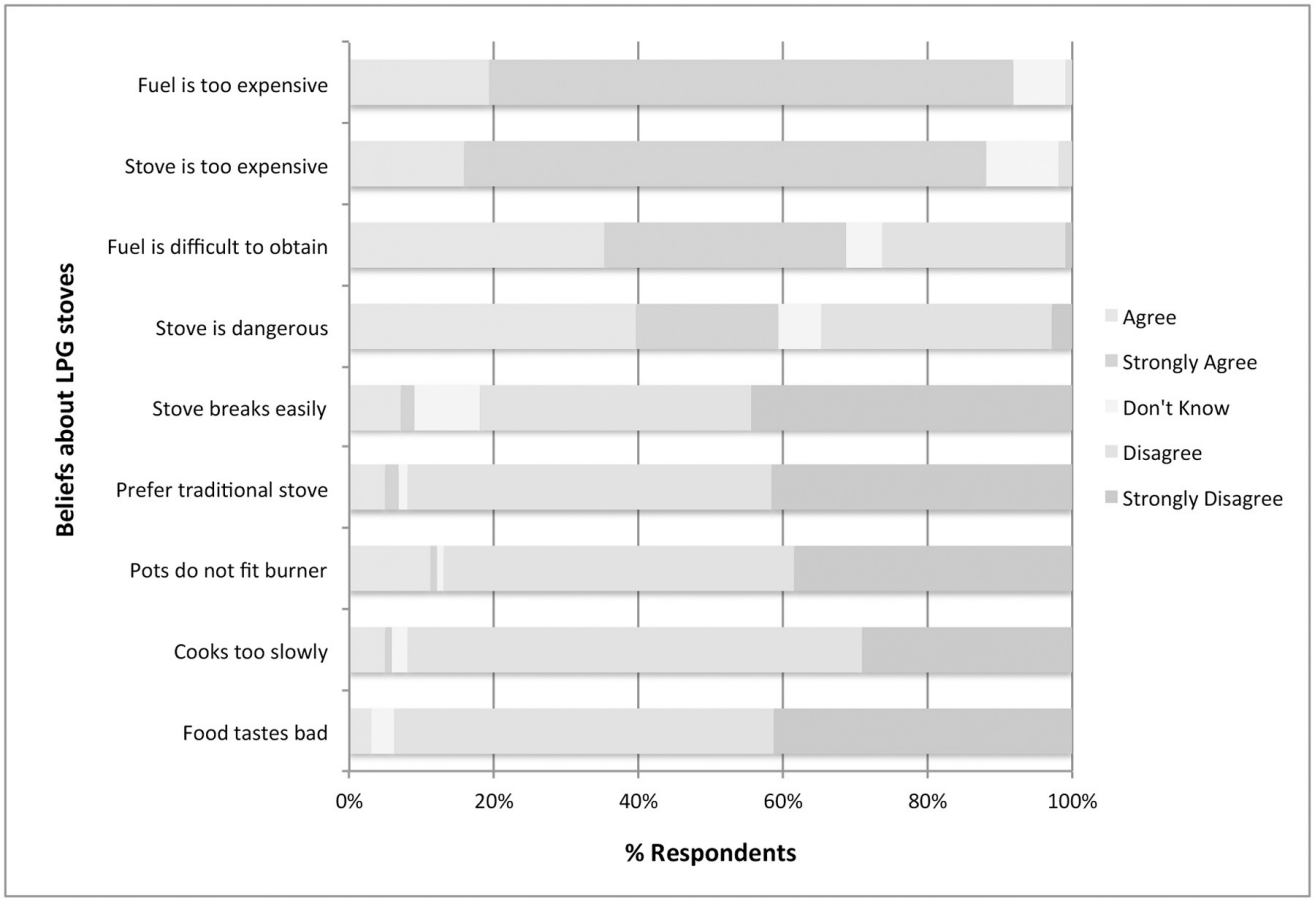
Likert scale presentation of perceptions of liquid propane gas stoves (LPG).

Respondents believed LPG stoves were efficient (n = 1024, 93%) and durable (81% disagreed that stoves break easily). A majority of respondents (n = 951, 86%) thought LPG stoves were well designed to accommodate typical pot sizes, however among the 287 households that already owned an LPG, 20% (n = 56) said the burner did not fit their pots. The flavor of food cooked with LPG stoves was deemed equal or better when compared to traditional stoves for the vast majority of respondents (86%, n = 951). Cost was the primary drawback of LPG stoves; 88% (n = 968) of respondents said the stove unit was too expensive and 92% (n = 1,008) felt the replacement fuel cost was too high. LPG stoves were perceived as dangerous by 60% (n = 657) of all respondents, however among those who already owned an LPG stove, 73% (n = 209) felt they were dangerous (Fig 5). Burns to children caused by cooking fires were reported in 190 (17%) households in the preceding year, however 30% (n = 57) of these occurred in households that owned LPG.

## Discussion

This investigation explores the cooking practices for typical households in the rural Niakhar area in the Fatick Health District of Senegal and the prevailing perceptions of traditional and clean stove technologies. Cultural practices and attitudes regarding cookstoves vary widely between geographic regions and even within individual countries. Accordingly, understanding a local community’s complex cooking tradition is essential for programs seeking to promote clean stove and fuel technology. Assessment of community cooking practices and stove preferences have not previously been described for rural Senegalese communities.

Households in this community adhered to cooking traditions commonly seen in resource-limited settings and described in similar Senegalese communities, including reliance on biomass fuels, indoor cooking patterns, and frequent use of more than one stove design also known as “stove-stacking” [23,24]. Biomass fuel use was ubiquitous and cooking in semi-enclosed or enclosed spaces was common. Type of biomass fuel used varied by season, with near universal wood burning during the rainy season being supplanted with combustion of dried dung cakes or crop residue during the dry months. Wood was typically collected during the dry months and stored for use during the rainy season. As other studies have shown, owning an LPG stove did not translate to use as a primary stove; less than 1% of LPG-owning households used this stove exclusively, or even as the primary device [24,25]. Stove-stacking with a traditional open-fire tripod stove was common practice, even for wealthier households.

Despite nearly universal use, traditional tripod stoves were not considered desirable in comparison to the alternative stove options. Women valued cooking efficiency, citing large cooking capacity and rapid heat generation as key stove features, and these are manifest characteristics of any traditional open-fire stove. However, low smoke emission was highly valued as well, more so than most other aspects, including fuel cost and availability. This preference to minimize smoke exposure may account for a substantial proportion of the dissatisfaction expressed for the stove they used most frequently in practice.

Our data would suggest that there is not a preference for the traditional stove motivating its widespread use. Rather, stoves that promised reduced smoke emissions, family health benefits, and reducing time spent collecting fuel, were substantially more desirable. Despite this, in many intervention studies of ICS, the uptake and sustained used of ICS falls short of supplanting the less desirable, but perhaps more utilitarian, traditional stove [12,26,27]. It may be that the tension between the practical necessity for rapid, reliable cooking and the less concrete desires such as reduced smoke exposure, skews heavily towards the practical considerations.

Previous data suggest that in some cultures, preserving conventional cooking practices with a traditional stove is valuable [28]. Women in our study did not endorse a flavor preference for food cooked over a traditional stove as compared to ICS or LPG, suggesting cleaner-burning stove alternatives can achieve the desired end result for traditional dishes. This is in contrast to other cultures where LPG use is curtailed, in part due to the opinion that it alters the taste of traditional meals [29].

LPG stoves satisfy the criteria for low smoke emissions, high cooking efficiency, and are perceived by members of the Niakhar community to have health benefits. Accordingly, when cost was removed from consideration, LPG stoves were cited as the most preferred stove type for one out of every three participants. However, the real-world implications of cost are substantial. The vast majority of participants, 92%, felt replacement LPG fuel canisters were too expensive and this was borne out by less than 1% of LPG-owning households actually using them as their primary cooking device despite an expressed preference for the clean technology over the traditional stove.

In regards to perceptions about LPG safety, there was some discrepancy between households that owned an LPG stove and those that did not. The majority of households that owned an LPG stove, nearly three in four, expressed safety concerns, while only about half of households without LPG viewed the stoves as dangerous. The majority of stove-related childhood burns occurred in non-LPG households.

Solar stoves were highly desirable in this community; second only to LPG, solar stoves were cited as the most desired stove for 28% of participants. However, the basis for this preference is unclear as solar stove technology is not used in this community and additional information about the technology and its benefits and drawbacks were not provided as a part of this survey. Although this specific community does not use solar stove technology, Senegal has made renewable energy, specifically solar energy, a national priority [30]. Since 2006, the Government of Senegal has partnered with multiple non-governmental organizations to trial, produce, and distribute a variety of solar cookstoves in communities throughout the country, including in a region adjacent to the study site [31–33]. The publicity surrounding these solar stove campaigns may explain the marked interest in solar that we observed in our study.

Important deficiencies of solar technology currently exist. In the randomized controlled solar stove trial conducted in Senegal, adoption was poor with stove usage plateauing at 19% at the 6 month follow-up [33]. The primary explanation for low usage was inadequate capacity for the stove; a stove with capacity to feed 6 adults is insufficient in a community where 60% of women cook for 11 or more people per day. Additional complaints included slow cooking time, poor durability, and inability to prepare specific traditional dishes that require intense heat. Given Senegal’s commitment to renewable fuels and the rapidly advancing field of solar technology, future research is warranted to explore community opinions and needs as they relate to solar stoves.

Perceptions of ICS were generally favorable, but preference for ICS design fell short of alternative stove designs, namely LPG. The ICS design most prevalent in the region is the *jambaar* style of clay-insulated metal stove that burns biomass fuel (Fig 2). However, other more technologically sophisticated ICS were also shown to participants, many of whom would not have had prior experience or knowledge of this stove style. Participants generally believed the broad category of ICS were durable, efficient, and culturally appropriate for traditional cooking methods, but did not express desire to own and use this type of stove. The lack of desire for ICS may partially be explained by the perception of prohibitive cost; the majority of respondents felt ICS were too expensive. However, cost alone did not explain the lack of interest.

Compared to the two most desirable stove types, LPG and solar, ICS still rely on biomass fuel which produces visible smoke emissions, regardless of degree of improved thermal efficiency certain ICS designs might confer. Both smoke emissions and time spent collecting fuel were two key problems that LPG and solar stoves might address for women in this community. An additional consideration is that the fuel chambers of ICS, often through an opening in the side of the stove, may demand fuel ‘preparation’ (i.e. cutting wood into smaller pieces) or make it difficult to add some types of common fuel (i.e. dried dung cakes or crop residue) to a burning fire. The time burden of preparing fuel for cooking may be an important consideration for women whose daily responsibilities leave little room for extraneous tasks.

There are limitations to this study. For some questions, particularly regarding stove and fuel preference (Table 1), up to 16% data missingness may indicate unwillingness by participants to discuss the topic or confusion about the topic being discussed. Self-report of primary stove used in household may not accurately reflect actual usage. Additionally, without objective measures of stove usage, the relative use of the many stoves owned by a household cannot be precisely assessed. The survey was administered in French, and while French is the national language of Senegal, many residents of Niakhar primarily speak Sereer. We employed highly trained field workers who were fluent in both languages; however misunderstanding due to language remains possible. Participants were asked about their opinions of certain stoves, some for which they may have had no first-hand knowledge or experience. These perceptions, even in the absence of real world experience with the stoves, are valuable because they describe a community’s pre-formed biases towards new stove technology. ICS come in a myriad of designs that utilize various fuels, and we asked questions about ICS generally, showing pictures of a representative sample of ICS stoves sold in Senegal. However, depending on exposure to these stoves, opinions may have been based on a specific stove design rather than the broader category of ICS. Finally, in the context of a preceding survey asking questions about child respiratory symptoms related to smoke [21], participants may have been inclined to report dissatisfaction with their traditional open-fire stove and show enhanced interest in alternative technologies.

## Conclusions

Fundamentally, stove intervention programs seek to reduce the negative health and environmental implications associated with biomass fuel combustion. Success is not predicated solely on technology, but rather, on behavior change. Adoption and proper use of cleaner stoves and fuel, with the concurrent rejection of traditional devices, is the ultimate goal and only possible through behavior change at the level of the individual and the community. Understanding the community motivations for change and the potential barriers to adoption is fundamental to any program seeking to reduce reliance on traditional, open-fire biomass stoves.

In this rural health district in Senegal, the traditional method of cooking over an open, biomass-fueled stove was a nearly universal practice. Stove stacking that incorporated the traditional stove was the rule, rather than the exception for households able to afford more than one stove. Despite the ubiquitous use of the traditional stove, it was the least desired type of stove when compared to all other available options. This widespread dissatisfaction with the traditional stove presents an opportunity for LPG and other clean fuel intervention programs. Indeed, LPG was the most preferred stove in our survey. However, possession of an LPG stove did not correlate with its use as the primary stove in a household. Women in our survey identified cost, limited cooking area and concerns about safety as drawbacks to LPG. These perceptions warrant further exploration if LPG adoption is to succeed in Senegal.
